# The ups and downs of Pax6 in neural stem cells

**DOI:** 10.1016/j.jbc.2023.104680

**Published:** 2023-04-05

**Authors:** Belal Shohayeb, Helen M. Cooper

**Affiliations:** The University of Queensland, Queensland Brain Institute, Brisbane, Queensland, Australia

## Abstract

Neural stem cells must rapidly adapt their transcriptional activity to the ever-changing embryonic environment. Currently, we have a limited understanding of how key transcription factors such as Pax6 are modulated at the protein level. In a recent issue of the JBC, Dong *et al* identified a novel posttranslational regulatory mechanism in which Kat2a-mediated lysine acetylation on Pax6 leads to its ubiquitination and ultimately its degradation via the proteasome pathway, thereby determining whether neural stem cells undergo proliferation or neuronal differentiation.

In the early vertebrate embryo, the neural stem cells (NSCs) lining the ventricles give rise to the extensive repertoire of neuronal cell types of the brain, retina, and spinal cord. Initially, NSCs (also known as radial glial progenitors) undergo symmetric self-renewing divisions to expand the progenitor pool and then switch to neurogenic divisions, generating specific neuronal subtypes in a temporally restricted manner (see Veeraval *et al*, 2020 (1) and references therein). The decision to exit the cell cycle is kept under strict spatiotemporal control by proliferative and neurogenic cues emanating from the developing neuroepithelium or cerebral spinal fluid (2). NSCs must therefore be able to rapidly adapt their transcriptional activity to the ever-changing embryonic environment.

The archetypal paired box transcription factor Pax6 was first identified in 1994, when loss-of-function mutations in the *Drosophila* ortholog *eyeless* were observed to severely reduce or eliminate the eye (3). Equivalent mutations in *Pax6* were subsequently found to be responsible for the small eye phenotype in the mouse and congenital abnormalities such as aniridia (absence of the iris) in humans (4). More recently, Pax6 has been shown to play a role in the regional patterning of the neocortex, the expansion of the cortical NSC pool, and the specification of some neuronal populations (4). Although much information is now available regarding the transcriptional regulation of the *Pax6* gene (5, 6), we still have only a poor understanding of how Pax6 is modulated at the protein level. Recently, Dong *et al*. uncovered a novel posttranslational regulatory mechanism that reduces Pax6 protein levels in NSCs (7). The central player in this new pathway is the histone acetyltransferase Kat2a (aka Gcn5; general control nonrepressed protein 5) known to promote transcription through chromatin remodeling (8). Dong *et al* have exploited both the zebrafish developmental model and rat NSC cultures to demonstrate that Kat2a-mediated lysine acetylation on Pax6 leads to its ubiquitination and ultimately its degradation via the proteasome (Fig. 1). As such, this study identifies a hitherto unappreciated role for histone acetyltransferases in the negative regulation of transcription factor activity.

**Figure 1:**
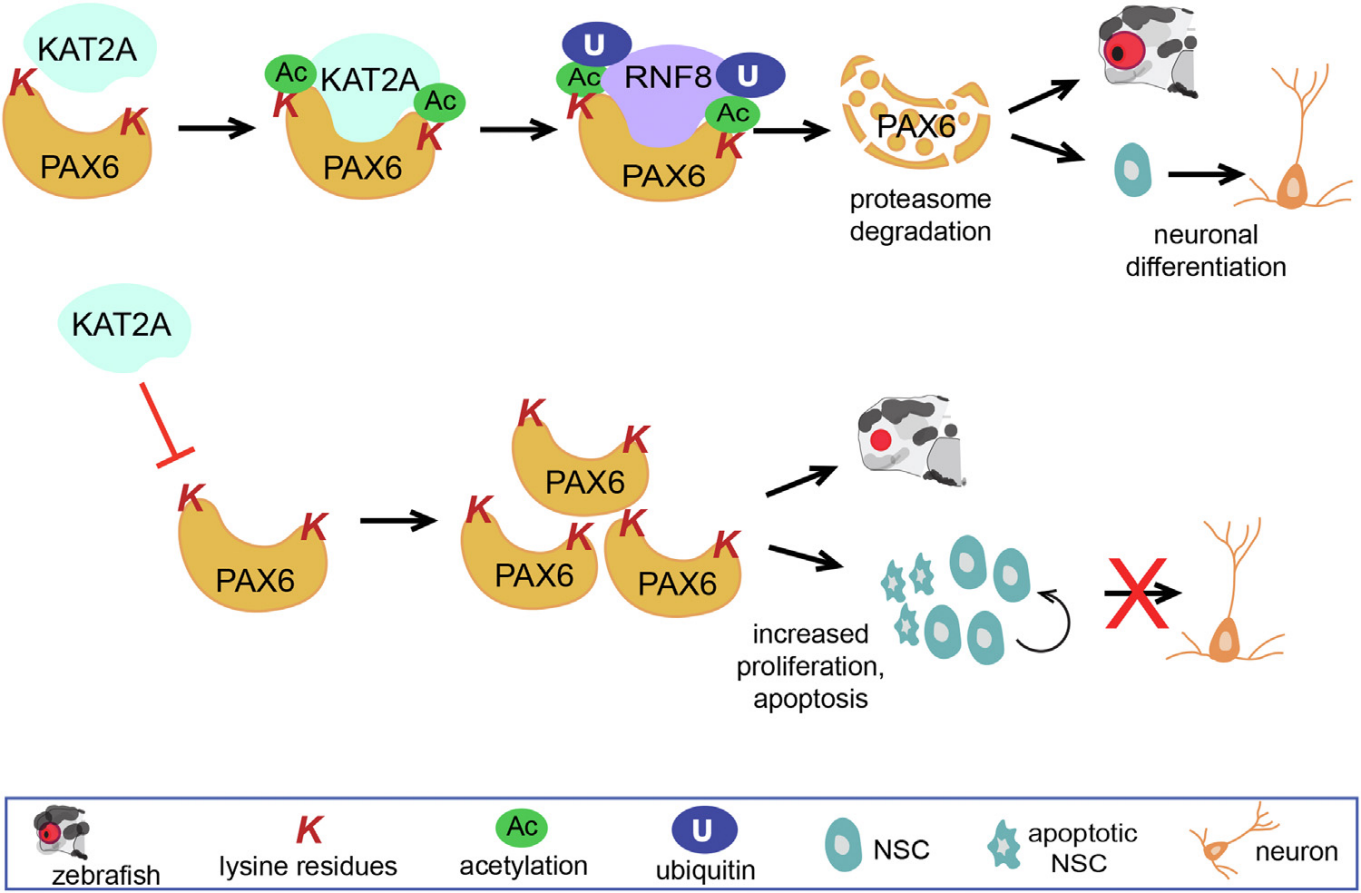
**Kat2a is a negative posttranslational regulator of Pax6 in zebrafish and murine NSCs.** Kat2a-mediated lysine acetylation on Pax6 leads to Rnf8 ubiquitination and proteasome degradation resulting in NSC cell cycle exit and neuronal differentiation. Inhibition of Kat2a–Pax6 interactions prevents neuronal differentiation and increases proliferation and apoptosis, leading to a severe reduction or elimination of the zebrafish eye. NSC, neural stem cell.

This story begins with the observation that the depletion of kat2a in zebrafish embryos using mRNA-blocking oligonucleotides (morpholinos) produced a small eye phenotype. Closer examination revealed that the NSCs that give rise to the retina exhibited excessive proliferation and subsequently disappeared due to the induction of programmed cell death (apoptosis). Recognizing that this phenotype closely resembled that seen in *Pax6* mouse mutants, the team then quantified pax6 in the mutant zebrafish eye and observed an increase in pax6 protein, but not *pax6* mRNA, strongly suggesting that kat2a reduces pax6 protein levels. This deduction was bolstered when overexpression of *pax6* in the zebrafish embryo reproduced the *kat2a* phenotype. Moreover, the small eye phenotype in kat2a-depleted embryos was fully rescued when pax6 protein production was blocked by morpholinos. Together, these findings provided strong genetic evidence that kat2a suppresses pax6 activity at the protein level.

Although the zebrafish is a valuable developmental model, it is less amenable to the in-depth biochemical interrogation required to map out molecular pathways. The team therefore turned to NSC cultures derived from the embryonic rat cortex, where they quickly confirmed that a decrease in Pax6 protein was correlated with an increase in Kat2a as NSCs underwent neuronal differentiation. The addition of the Kat2a inhibitor MB-3 prevented neuronal differentiation, increased NSC proliferation and apoptosis, and maintained Pax6 protein levels across the differentiation period. The authors then embarked on an elegant biochemical study to dissect the molecular pathway involved. Using coimmunoprecipitation assays, they demonstrated a direct interaction between Kat2a and the N-terminus of Pax6. These assays yielded three additional key observations: Pax6 became increasingly ubiquitinated on lysine residues as NSCs differentiated; proteasome inhibition stabilized Pax6 protein levels; and thirdly, Kat2a-dependent acetylation of Pax6 lysine residues increased as differentiation proceeded. As ubiquitination is a major driver of protein degradation via the proteasome complex (9), this suggested that Kat2a-mediated lysine acetylation may mark Pax6 for ubiquitination and proteasomal degradation. Indeed, MB-3 inhibition of Kat2a reduced Pax6 ubiquitination. This hypothesis was unambiguously confirmed following the identification of lysines 75 and 264 as the primary acetylation sites using mass spectrometry. Clever mutagenesis experiments were performed where K75 and K264 were converted to glutamine to mimic acetylation or to arginine to prevent acetylation. As predicted, expression of the glutamine mutants, but not the arginine mutants, led to a substantial reduction in Pax6 protein.

The final piece of the puzzle was the identification of the ubiquitin ligase responsible for targeting Pax6 for degradation. The E3 ligase Rnf8 (Ring finger protein 8) was considered the likely culprit, as it was expressed in differentiating NSCs and could be coprecipitated with Pax6. Furthermore, knockdown of Rnf8 using siRNAs prevented Pax6 degradation. To complete the story, morpholino knockdown of Rnf8 in the zebrafish embryo recapitulated the Kat2a and Pax6 small eye phenotype. In conclusion, this study identified new roles for Kat2a and Rnf8 as negative posttranslational regulators of Pax6 in NSCs and as such, suggests that they are likely to play important roles in the decision to undergo proliferation or differentiation.

Mitotic NSCs exist in a plastic state where they maintain their proliferative capacity by expressing progenitor-specific transcripts, while concomitantly synthesizing neurogenic mRNAs (reviewed in Shohayeb et al. (10)). The activity of the proliferating *versus* differentiating transcriptional networks must therefore be finely balanced. Although recent technological advances in single-cell profiling have opened a fascinating window into the complex regulatory pathways governing transcription and epigenetics, less is known about posttranslational mechanisms. The discovery by Dong *et al* that Kat2a and Rfn8 target Pax6 for proteasomal degradation provides a new mechanism through which transcription factor activity can be rapidly silenced. Two intriguing questions immediately come to mind. How many other transcription factors are subject to the Kat2a–Rfn8 degradation pathway? More broadly, is this a global mechanism through which NSC transcription factor networks are synchronized in response to environment cues?.

## Conflicts of interest

The authors declare they have no competing interests with the contents of this article.
